# Developing an Algorithm for Discriminating Oral Cancerous and Normal Tissues Using Raman Spectroscopy

**DOI:** 10.3390/jpm11111165

**Published:** 2021-11-09

**Authors:** Mukta Sharma, Ming-Jer Jeng, Chi-Kuang Young, Shiang-Fu Huang, Liann-Be Chang

**Affiliations:** 1 Department of Electronic Engineering, Chang Gung University, Taoyuan 333, Taiwan; d000016796@cgu.edu.tw (M.S.); liann@mail.cgu.edu.tw (L.-B.C.); 2 Department of Otolaryngology-Head and Neck Surgery, Chang Gung Memorial Hospital, Linkou 244, Taiwan; shiangfu.huang@gmail.com; 3 Department of Otolaryngology, Head and Neck Surgery, Chang Gung Memorial Hospital, Keelung Branch, Keelung 204, Taiwan; rioriorioman@gmail.com; 4 Department of Public Health, Chang Gung University, Taoyuan 333, Taiwan; 5 Green Technology Research Center, Chang Gung University, Taoyuan 333, Taiwan

**Keywords:** oral cancer, Raman spectroscopy, PCA-LDA, PLS-LDA, cryopreserved tissue

## Abstract

The aim of this study was to investigate the clinical potential of Raman spectroscopy (RS) in detecting oral squamous cell carcinoma (OSCC) in tumor and healthy tissues in surgical resection specimens during surgery. Raman experiments were performed on cryopreserved specimens from patients with OSCC. Univariate and multivariate analysis was performed based on the fingerprint region (700–1800 cm${}^{-1}$) of the Raman spectra. One hundred thirty-one ex-vivo Raman experiments were performed on 131 surgical resection specimens obtained from 67 patients. The principal component analysis (PCA) and partial least square (PLS) methods with linear discriminant analysis (LDA) were applied on an independent validation dataset. Both models were able to differentiate between the tissue types, but PLS–LDA showed 100% accuracy, sensitivity, and specificity. In this study, Raman measurements of fresh resection tissue specimens demonstrated that OSCC had significantly higher nucleic acid, protein, and several amino acid contents than adjacent healthy tissues. The specific spectral information obtained in this study can be used to develop an in vivo Raman spectroscopic method for the tumor-free resection boundary during surgery.

## 1. Introduction

Oral cancer ranks sixth among other types of cancer globally [1]. Smoking, consuming alcohol, chewing tobacco, and chewing betel quid are all closely related to oral cancer. Approximately 650,000 people are diagnosed with oral cancer and about 300,000 deaths are reported annually [2]. Oral cancer is the fifth leading cause of mortality in Taiwan, and the fourth leading cause of death among men. [3]. Taiwan has the highest crude incidence rate of oral cancer in the world, at 32.46 per 100,000 people [2]. Betel nuts are easily available in Taiwan and about 86% of Taiwanese oral cancer patients consume betel nuts on a regular basis [4]. The oral cancer mortality rate is high because of late-stage cancer diagnosis, at which stage the disease has progressed, and the survival rate drops to 15–50% [5]. Oral potentially malignant disorders (OPMDs), which are inclined toward malignant transformation, can be prevented if pre-malignancy is detected early and treated promptly [6]. Hence, early-stage diagnosis is an essential step in improving disease-free survival rates. In practice, oral cancer is observed by clinicians using a conventional oral examination (COE), with patients further referred to a biopsy, followed by histologically proven analysis for the definitive diagnosis [7]. Under incandescent light, COE performs poorly and is insufficient for risk assessment [8]. Oral cancer is diagnosed via a biopsy, which is the gold-standard method. Biopsy, on the other hand, is an invasive procedure that causes patient discomfort and requires skill, as well as expensive laboratory facilities that are only available in high-resource environments. Hence, to reduce the number of unnecessary biopsies and to enable the early-stage detection of oral cancer, several diagnostic tools have been developed at the clinical and molecular levels to assist in evaluating OPMDs in a non-invasive manner. Many non-invasive techniques, such as vital staining techniques (e.g., toluidine and methylene blue), optical imaging devices (fluorescence, chemiluminescence, Raman spectroscopy (RS), and optical coherence tomography), and exfoliated cytology tools, have been developed to assist in the early detection of OPMDs [9]. Previously, one optical imaging device—VELscope (which works on autofluorescence phenomena)—was used for oral cancer detection with a quantitative analysis method to classify autofluorescence images to improve the use of VELscope [10]. This quantitative analysis approach is able to distinguish between normal, premalignant, and malignant cells. RS is an optical technique that is most widely used for the non-destructive characterization of molecules, and no tissue labeling is needed [11]. Multivariate analysis is still needed to identify the most diagnostically significant characteristics in the spectrum dataset and to investigate biochemical changes further. Using various preservation procedures and analytical methods, many studies have proven the efficacy of RS in discriminating between normal and malignant or between normal, premalignant, and malignant types of oral mucosa [12,13,14,15,16,17,18]. The efficacy of RS (an optical diagnostic technique) was demonstrated using an excitation wavelength of 532 nm for subsite-wise (tongue, buccal mucosa, and gingiva) oral cancer detection [19]. In our previous study [20], we proposed a novel quantitative method for detecting oral cancer using two different optical techniques.

In this study, patients suffering from oral cancer were investigated. A cryopreservation technique was used to preserve the tissue sample, which can prevent the alteration of the structure and morphology of the tissue. Tissue samples were collected and tested under RS. These measurements were combined with histological classifications to evaluate the performance of machine learning models (PCA–LDA and PLS–LDA). The objective of this study was to prove the potential of RS in differentiating OSCC from normal tissues by developing ex vivo tissue classification models based on spectral data from individual tissue structures. The validation of the models was performed using an independent dataset.

## 2. Methods and Materials

### 2.1. Patients and Samples

The Chang Gung Medical Foundation’s Institutional Review Board (IRB) approved this study (IRB No: 201800420B0). The registered participants gave their written and informed consent to participate in this study, which was carried out in the Department of Otolaryngology–Head and Neck Surgery. All specimens and pathological reports were collected at Chang Gung Memorial Hospital for analysis. A total of 131 tissue samples were collected from 67 patients who had undergone a surgical resection because of OSCC, and were tested under RS. Sixty-seven tumorous samples were collected from 67 patients, and the other 64 samples (taken from a site adjacent to the tumor) contained only normal tissue and were harvested (Table 1). Surgical resection specimens from normal-appearing mucosa adjacent to the tumor were taken 15–30 minutes after surgery, whereas tumor samples were acquired immediately after surgery. The cryopreserved samples were freshly cut and stored in liquid nitrogen (N${}_{2}$) at −80 °C to retain their morphology until usage. Each tissue sample had five spectra taken at different positions. Thus, 655 spectra were acquired from the two types of tissue samples.

### 2.2. Development of Tissue Classification Models Using the Training Data Set

The spectra of 80 tissue samples, randomly chosen from 67 patients, were used to develop tissue classification models. The 80 samples were collected from 44 tumor patients, and the other 36 samples (taken from a site adjacent to the tumor) contained only normal tissue during surgery. In this study, two tissue classification models were developed: (1) a PCA–LDA model tumor versus normal tissue (adjacent to the tumor) and (2) a PLS–LDA model tumor versus normal tissue. For developing the tissue classification models, PCA and PLS were used, followed by LDA.

### 2.3. Validation of Tissue Classification Models Using a Validation Data Set

The final tissue classification models were validated using the remaining spectral database from 51 tissue samples (28 normal and 23 tumors) that contained 23 different patients not included in the training set. In a receiver operator characteristic (ROC) study, the true positive rate (sensitivity) was plotted against the false positive rate (1-specificity) for various values of the discrimination threshold to estimate the discriminative ability of the tissue classification models. The ROC curve was used to select a threshold for a classifier that maximizes true positives and minimizes false positives. It also allows us to evaluate the classifier’s performance across its whole operating range [21]. The area under the ROC curve (AUC) measures the discriminatory power of the tissue classification model. AUCs of >0.9 are considered to have strong discriminative power, AUCs of 0.8 to 0.9 are considered good, AUCs of 0.7 to 0.8 are considered moderate, and AUCs of 0.6 to 0.7 are considered poor [22].

### 2.4. Pre-Processing of Spectra

MATLAB (R2018a, MathWorks, Natick, MA, USA) was used to process and analyze the data. First, to reduce interference, a Savitsky–Golay filter was employed to smooth the recorded spectra. Then, after the baseline correction, normalization was performed using the area under the curve (AUC) technique to eliminate the data redundancy [19]. Finally, PCA and PLS were applied to the normalized spectrum from 700 to 1800 cm${}^{-1}$.

### 2.5. Instrumentation

The RS instrument (ProTrusTech Co. LTD, New Taipei City, Taiwan) was employed, comprising a laser with a wavelength of 532 nm and a laser power of 126 mW as an excitation source. Spectral acquisition proceeded as follows: the laser power was 6.3 mW to 12.6 mW, the integration and acquisition times were 5 s and 15 s, respectively, and the average value of spectrum was three. The manufacturer specified a spectral resolution of 1 cm${}^{-1}$.

### 2.6. Univariate and Multivariate Analysis

The mean-normalized spectrum was analyzed using ROC curves, and two multivariate statistical methods, PCA–LDA and PLS–LDA, were employed. The primary idea of using these classifier models is to reduce redundancy and noise from a huge dataset by means of the PLS and PCA methods and to use the dimension reduction features as the input of the LDA algorithm. PCA is a statistical approach for reducing dimensions and producing new principal components. In the PCA technique, importance is given to the predictive variables, omitting the relationship between each predictive variable and the target variable. PLS helps in achieving this balance by building standardized linear combinations of predictive variables to capture as much information as possible. It also establishes a relationship between the predictive and target variables [23]. The LDA classifier model was used to study the boundary between the classes and the probabilities of classification. This model maximizes the ratio of “between-class variance” to “within-class variance”. The LDA classifier creates a linear boundary by assuming a common covariance matrix. The classifier models were optimized using a training dataset to evaluate the classified results, and a test data set was used to assess their performance. First, to reduce the dimensionality of the gathered data, an unsupervised multivariate analysis method, such as PCA, was used. Second, the supervised classification algorithm PLS method was used. The latent variables (LVs) are rotated in PLS–DA (discriminant analysis) to obtain maximal group separation. As a result, rather than relevant variations in the dataset, the LVs analyze diagnostically relevant variations [24]. Before PCA and PLS analysis, the Raman data were pre-processed. The first five principal components (PC1 to PC5) accounted for up to 98% of the variance, as evaluated by PCA, whereas in the PLS model, the first five factors showed a high correlation coefficient of 0.8 between the X and Y scores. They were fed into the LDA classifiers. This study classified normal and malignant tissues broadly. To remove heterogeneity, each sample’s average spectrum was evaluated. Scores of factors 1, 2, and 3 were selected for both models to generate a three-dimensional scatter plot with a decision boundary curve.

## 3. Results and Discussion

There were a total of 131 ex-vivo Raman mapping experiments conducted on cryopreserved specimens from 67 patients who had undergone surgical resection for OSCC. Of these 131 samples, 80 samples (from 44 patients) were used to develop the method for detecting tumors (which was called the training set). The remaining 51 samples (from 23 different patients) were used to validate the model (the testing dataset).

### 3.1. Spectral Analysis

**Figure 1:**
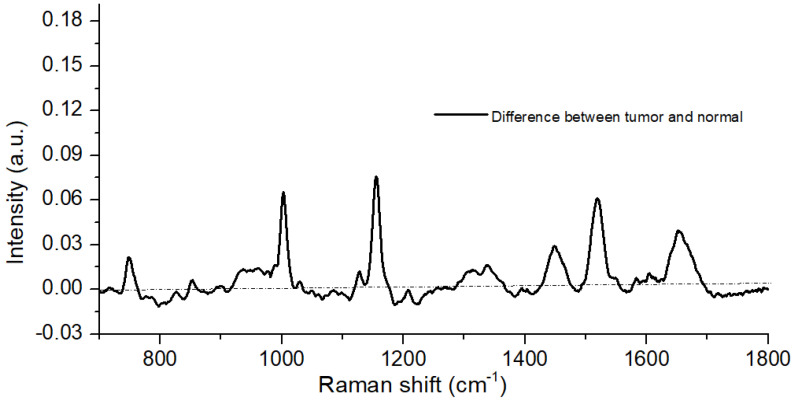
Difference spectra were calculated from the mean spectra (tumor minus normal).

The mean Raman spectra (131 samples) of each group were obtained for each model, to visually analyze the differences between the groups. On the difference spectra between tumor tissue and normal tissue, these differences can be seen explicitly in Figure 1. The positive peaks were presented due to tumor tissues, and negative peaks appeared due to normal tissues. Positive peaks dominated the difference graph for almost all of the metabolites found in our study, indicating that most of the metabolite levels were higher under tumorous conditions. The negative peaks at 1064, 1168, and 1220 cm${}^{-1}$ in the normal spectrum and positive peaks at 1004, 1123, 1156, 1302, 1450, 1516, and 1650 cm${}^{-1}$ in the tumor spectrum caused the great variation in these spectra.

**Figure 2:**
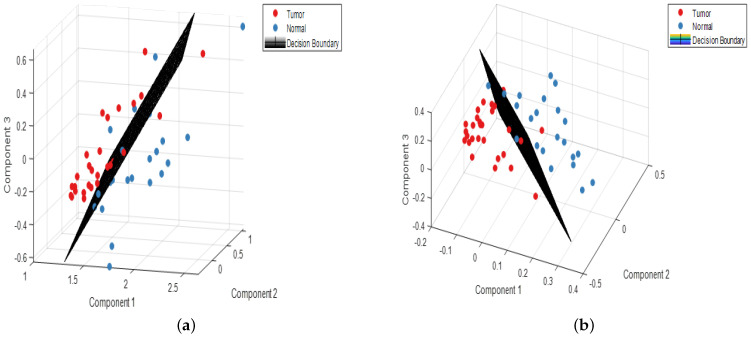
(**a**) PCA-LDA and (**b**) PLS- LDA decision boundary curves in 3D.

**Figure 3:**
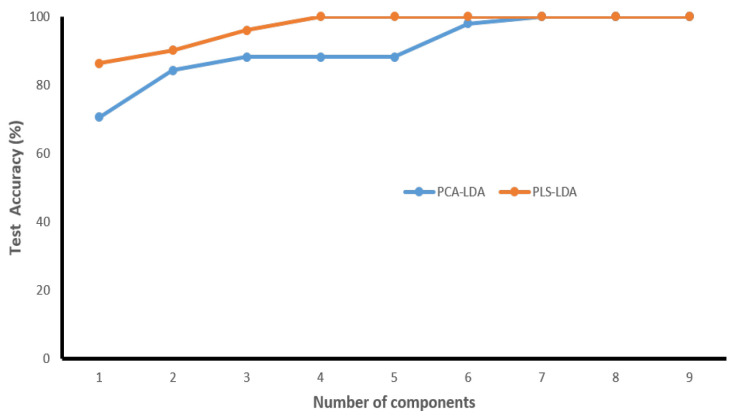
Accuracy rates versus number of components on PCA-LDA and PLS-LDA in the oral cancer dataset.

**Figure 4:**
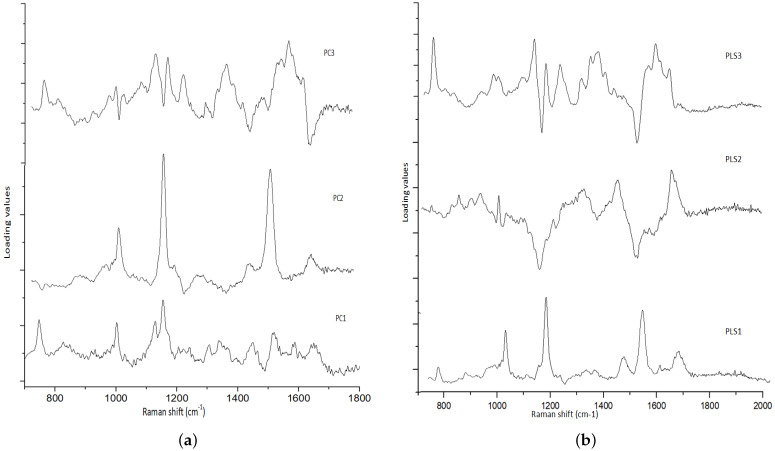
Loading plot of (**a**) PC components (PCs) and (**b**) PLS components (LVs) in each model calculated from the Raman spectra of oral tissues.

In our study, we observed that malignant or tumor samples had higher peaks than normal samples. Proteins, nucleic acids, amino acids, carbohydrates, and lipids abounded in the fingerprint region (700 cm${}^{-1}$ to 1800 cm${}^{-1}$) in biological tissues. The use of these main distinguishing spectral features is very plausible for differentiating between normal and malignant tissues. It has been verified in the literature that normal tissue spectral peaks are dominated by lipids, whereas malignant tissue peaks are dominated by proteins [19]. The assignments observed in Raman spectra modes are gathered in Table 2. The spectra of normal tissues were dominated by peaks at 1004, 1156, 1339, 1450, 1523, and 1656 cm${}^{-1}$, whereas malignant or tumor tissue samples were dominated by peaks at 1064, 150, 1168, and 1220 cm${}^{-1}$. It can be seen both kinds of tissues have carotenoid peaks at 1518 cm${}^{-1}$ and 1156 cm${}^{-1}$, and the tumorous tissues had more intense carotenoid peaks than normal tissue. In one study [25], peaks at 1004, 1518, and 1156 cm${}^{-1}$ were exclusively seen in normal tissues, whereas they were absent in tumor tissues. In one study [26], these peaks (1006, 1156, and 1518 cm${}^{-1}$) were seen in the carcinoma Raman patterns during the investigation (and were much less intensive in healthy tissues). The occurrence of these peaks (1156 and 1518 cm${}^{-1}$) in our analysis was due to mixed signals, with carotenoid and protein contributions. The peak at 1004 cm${}^{-1}$ is attributable to the protein’s phenylalanine characteristics. The reason for not bleaching the carotenoid signal in our investigation was that the resonance effect caused a significant amplification of the carotenoid bands at 532 nm (the incident radiation wavelength occurs within the range of electronic absorption of carotenoids) [27]. The scores of factors (components) provide quantitative information on the biomolecules present, and the information obtained from the scores can be used to discriminate between the various groups present in a huge dataset. Using the scores of factors 1, 2, and 3, classification was performed among the two groups. The calculated PCA–LDA and PLS–LDA data are shown in Figure 2a,b, with a 3D decision boundary curve of three consecutive PCs and PLS components, respectively. The decision boundary classified tumor tissue and normal tissue. The tumor and normal classes are represented by solid red and blue dots, respectively. It is justified that the classification accuracy of both models increased with the number of components and tended to be stable when reaching a certain value (Figure 3). It has been observed that the first three PCs accounted for 98% of all the spectral variance (PC1 = 89.2%, PC2 = 4.9%, PC3 = 2.4%, PC4 = 1.5%, PC5 = 0.4%). The loadings of the PCs, in general, explain the variability of each PC as a function of wave-number and provide qualitative information about the biomolecules present in the tissue samples. The loadings of PC1, PC2, and PC3 and PLS1, PLS2, and PLS3 are shown in Figure 4. The most intensive bands at 749, 1004, 1156, 1450, 1517, and 1650 cm${}^{-1}$ are calculated for components 1 and 2. The plot of PC loadings (Figure 4a) indicated that PC1 presented spectral characteristics of proteins and lipids (peaks at 749, 1004, 1123, 1156, 1302, 1450, and 1517 cm${}^{-1}$); PC2 indicated the spectral characteristics of phenylalanine and amide I protein (peaks at 1004, 1156, 1517, and 1650 cm${}^{-1}$); PC3 showed the main features of lipids (main peaks at 848, 1128, 1168, 1302, and 1450 cm${}^{-1}$). Figure 4b shows the three significant PLS components’ (LVs) loading for the Raman spectral dataset. The loading of PLS1 contained the following specific Raman peaks from proteins: 749, 1004, 1156, 1450, 1517, and 1650 cm${}^{-1}$; The loading on PLS2-captured Raman peaks was mainly associated with proteins and lipids, as evidenced by the peaks at 1450, 1650, and 1064 (cm${}^{-1}$); The loading on PLS3 indicated the spectral characteristics of tryptophan (748 cm${}^{-1}$), lipids (1220 cm${}^{-1}$), and protein (1004 cm${}^{-1}$).

### 3.2. Characteristics of the Validation Set

An independent dataset was used to validate the tissue classification models. This validation set comprised 255 Raman spectra obtained from 51 samples from 28 patients excluded from the training set. Of these 51 tissue samples, 23 contained OSCC and normal tissue structures, whereas eight contained normal tissue structures only.

### 3.3. Validation of the Classification Models

**Figure 5:**
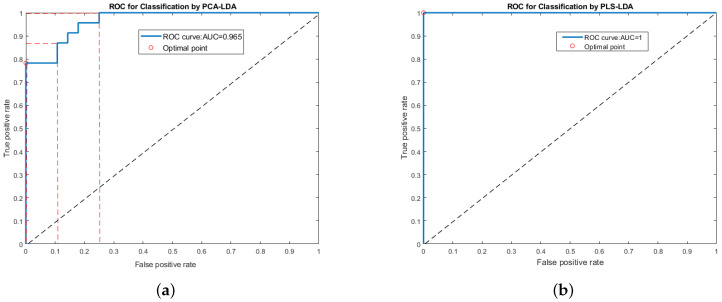
ROC curves of the two-group study for all cut-off values using (**a**) PCA-LDA and (**b**) PLS-LDA classifier models.

This study presents the results obtained from the validation dataset. To distinguish tumor tissue from normal tissue, a PCA–LDA model was first developed. To avoid over-fitting, the number of PCA components was employed in less than half of the minimal sample classes [29]. This model employed five PCs and achieved a classification accuracy of 90.2% as shown in Table 3. The ROC analysis showed an AUC of 0.965, as shown in Figure 5a. Specificity reached 100%, with a sensitivity of 78.3%. The PLS–LDA model was then employed to differentiate between tumor and normal tissue. The number of components included in PLS–LDA was chosen by increasing the number until the algorithm could correctly predict the pathology group of the spectra [30]. This model consisted of five PLS components and had a classification accuracy of 100%. The ROC curves for the PLS–LDA model are presented in Figure 5b, which shows that the maximum value of AUC is 1. Specificity was 100% at a sensitivity of 100% (Table 3). Both the PCA–LDA and PLS–LDA models were able to discriminate between cancerous and non-cancerous tissues, whereas the PLS–LDA model had an area closer to one. Using the ROC curve, we can see that for the PCA–LDA model, the sensitivity can be increased from 78% to 100%, and the specificity can be increased from 75% to 100%. This may be good for the proposed clinical applications.

We have demonstrated that RS can be a useful tool for predicting the presence of tumorous tissue in cryopreserved samples. Two models (PCA–LDA and PLS–LDA) were analyzed for stratified OSCC and non-tumorous tissues. The PCA and PLS data were calculated for healthy and OSCC patients in the 700–1800 cm${}^{-1}$ fingerprint region. The spectral data were analyzed based on the first five principal/PLS components, and results were presented on the 3D decision boundary curves, using three components (PCA/PLS). Using five PCs and a PCA calculation for the spectral fingerprint region, it was possible to determine 98% of the spectral variance between healthy and malignant tissue. The PLS–LDA model is more suitable than PCA–LDA for spectral data [31]. Only a classical discriminant method (such as LDA) is not feasible for spectral data [32], although PCA could be used to recover the pitfalls of LDA associated with high-dimensional spectral data. However, previous studies have confirmed the better performance of PLS–LDA than PCA–LDA [24,30,33,34]. PLS–LDA is the most effective statistical tool for multivariate analysis. ROC curves were used to investigate the two-group models’ clinical potential. These curves make it possible to choose the optimal costs and benefits for a certain application. Using a ROC curve, we can optimize the model’s performance toward detecting true positives, while retaining the number of false positives at an adequate level. The AUC allows us to estimate the correlation accuracy, specificity, and sensitivity at any degree of significance probability. Therefore, the ROC for all cut-off values and the correlation was determined and presented for the PCA–LDA and PLS–LDA methods. The determined correlation accuracy based on the presented data for PCA–LDA was 0.965 (96.5%), whereas it was 1 (100%) for the PLS–LDA method. The PCA–LDA and PLS–LDA models revealed that most of the biomolecular information from tissues and cells is crucial for distinguishing tumorous tissues from healthy tissues. These models can be differentiated using the different components of Raman biochemical and biomolecular features. Previously, in vitro tissue classification models have been developed and validated using RS (using only tongue subsites with few samples) [35]. In this study, the total maximum number of samples from different subsites was tested and we attempted to acquire an elevated sensitivity and specificity using a different classification model. The peak present at 749 cm${}^{-1}$ is a specific assignment of the C-C/C=C stretching mode of tryptophan associated with the amino acid. The proline, hydroxyproline, and ring breathing modes of tyrosine are represented by the band at 848 cm${}^{-1}$. A short and weak band was present at 1064 cm${}^{-1}$ and was attributed to the C-C skeletal stretching of lipids (higher in normal tissue). The peak present at 1123 cm${}^{-1}$ belongs to the DNA assignment of nucleic acid [36]. In this study, it was found that the important contributions of phenylalanine, tryptophan, amide I, amide III, and nucleic acid demonstrated increased peak intensities in tumor tissues. However, the contributions of lipids and carbohydrates were higher in normal tissues. Although peaks at 749, 848, and 1004 cm${}^{-1}$ could be associated with amino acid components, 1123 cm${}^{-1}$ with nucleic acids, and 1302 cm${}^{-1}$ with amide III, which were relatively strong in the tumor tissue, peaks at 1064, 1168, and 1220 cm${}^{-1}$ could be associated with higher lipid features. Strong peaks of proteins were present due to amide I (1650 cm${}^{-1}$) and CH2 deformation (1450 cm${}^{-1}$). Spectral contributions from the resonant beta-carotene features (1517 cm${}^{-1}$) played an important role in this classification due to their alteration during cancer. Using the RS, the biochemistry of cancerous and normal oral tissues can be analyzed rapidly. In this study, we used three subsites (the tongue, buccal mucosa and gingiva) in one frame, although different subsites were known to vary in regard to prognosis, metastases to lymph nodes, aggressiveness, and overall survival rate. In the future, we plan to investigate a large number of samples in the oral cavity with subsite-wise classification.

## 4. Conclusions

In this study, we validated RS-based ex-vivo tissue classification models for discrimination between OSCC and normal tissue. The PLS–LDA model has greater classification efficiency than the PCA–LDA model due to the supervised nature of its methodology, and the PLS technique is more efficient than the PCA method for the reduction of dimensions. It can also be beneficial in order to achieve an adequate boundary for the tumor-free resection margin during surgery. The results of our study highlight the feasibility of RS-based analytical approaches for favorable monitoring assessments and pathological diagnostic studies.

## Figures and Tables

**Table 1:** Illustration of tested tissue samples.

Subsites	Tongue	Buccal Mucosa	Gingiva	Total
Tumor	20	30	17	67
Normal	18	29	17	64

**Table 2:** Band assignments of Raman peaks [12,17,19,28].

Raman Bands (cm${}^{-1}$)	Compound/Assignments
749	Symmetric Breathing of Tryptophan (Protein Assignment)
848	Tyrosine (protein assignment)
1004	Phenylalanine (ring breathing mode)
1064	Skeletal C-C stretch of lipids
1123	(C-N), proteins (protein assignment)
1156	C-C(and C-N) stretching of proteins/ carotenoid
1168	Lipids
1220	=CH bending (lipids)
1302	Amide III (protein)
1450	CH2 bending in proteins and lipids
1517	beta -carotene or porphyrin
1650	In normal tissue due to C=C of lipids and in tumor tissue due to
	Amide-I-proteins

**Table 3:** Validation set misclassifications and performance tables of PCA-LDA and PLS-LDA models.

Dataset	Confusion Table			Performance Parameters		
PCA-LDA	Normal	Tumor	Total	Accuracy (%)	Sensitivity (%)	Specificity (%)
Normal	28	0	28	90.2	78.3	100
Tumor	5	18	23			
PLS-LDA	Normal	Tumor	Total	Accuracy (%)	Sensitivity (%)	Specificity (%)
Normal	28	0	28	100	100	100
Tumor	0	23	23			

## Data Availability

Not applicable.
